# Affective-Motivational Task Content and Stimulus Size Modulate Cognitive Control in Task Switching

**DOI:** 10.1027/1618-3169/a000665

**Published:** 2026-06-17

**Authors:** Leif E. Langsdorf, Josephine Gümbel, Viktoria Maydych, Torsten Schubert

**Affiliations:** ^1^ Institute for Psychology, Martin Luther University Halle-Wittenberg, Halle, Germany

**Keywords:** food stimuli, cognitive control, visual salience, affective-motivational dominance

## Abstract

**Abstract:** We investigated how cognitive control is modulated by the affective-motivational properties of food stimuli and their visual size. Prior research showed reduced switch costs – i.e., smaller costs in switch than in repetition trials – for food-related tasks compared to food-unrelated tasks. This raises the question of whether such effects are driven by the affective-motivational salience of food stimuli, by their visual salience due to larger size, or by an interaction of both factors. We addressed this using a task-switching paradigm in which participants alternated between a food categorization task (sweet/savory) and a digit categorization task (odd/even). The food stimulus was superimposed on the digit stimulus, and visual size was inverted compared to earlier studies, making the digit visually salient. Despite its smaller size, the food task still yielded reduced switch costs, suggesting that this effect cannot be attributed to visual salience. However, a direct comparison with prior findings revealed that visual size modulates the magnitude of the switch-cost asymmetry. This suggests that food stimuli improve task-set reconfiguration, but that visual salience likely plays a modulatory role. These findings indicate the need to consider motivational and perceptual factors in cognitive control research.


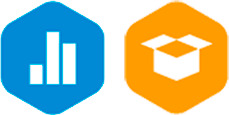


Humans possess the ability to flexibly switch between several tasks. Over the past decades, numerous studies have investigated this ability and identified the underlying control processes (Kiesel et al., 2010). Indeed, research has made significant progress in defining the control processes for flexibly switching between tasks, while lately the role of affective-motivational task content in modulating these processes has been tackled. In this context, food stimuli represent a relevant yet understudied stimulus category. They are ubiquitous in everyday lives, highly relevant for behavior, and capable of eliciting motivational-affective responses that are of interest in psychological research. Although a few studies have begun to examine how food stimuli modulate control processes, this line of research is still in its early stages, requiring further investigation (Maydych et al., 2024; Rutters et al., 2015). The present study examined how food stimuli and their visual size modulate cognitive control when participants are required to switch between a food-related task and a food-unrelated task.

## Task Switching

Task switching is widely used to investigate cognitive control. In this paradigm, participants are asked to repeat (the same task compared to the previous trial) or switch (perform a different task compared to the previous trial) between two sensory–motor reaction time (RT) tasks in quick succession. This usually results in switch costs, i.e., higher costs associated with switching compared to repeating the task. These costs are usually explained by a combination of top-down and/or bottom-up accounts (Kiesel et al., 2010). Most previous studies focused on the general mechanisms involved in switching between tasks; the potentially specific influence of affective-motivational task properties on cognitive control has received little attention, which warrants further investigation.

## Food Stimuli in Task Switching

Recently, Maydych et al. (2024) investigated how cognitive control is modulated by combining food stimuli with nonfood stimuli. The authors asked participants to perform both alternating runs with a predictable task sequence of AABB and a voluntary task-switching paradigm. On each trial, participants were shown a small digit superimposed on a larger food stimulus, and they either classified the digit as odd/even or the food stimulus as sweet/savory. Furthermore, the motivational state of participants was manipulated by assigning them either to a fasting or to a control condition. Across paradigms, participants showed greater switch costs for the digit task compared to the food task. In the alternating runs paradigm, this switch-cost asymmetry was further increased under fasting compared to the control condition. The authors suggested that the motivational state and the motivational salience of the food stimulus jointly modulated switching processes. It was assumed that the food stimulus led to a rapid activation of the food task-set within working memory, which enabled an improved task-set reconfiguration (Rutters et al., 2015). This assumed rapid activation results in a reduction of switch costs for the food compared to the digit task. However, as an alternative explanation, it is conceivable that the switch costs were not primarily modulated by motivational saliency of the food stimulus, but rather by its increased size compared to the digit stimulus. If that were the case, then the results would stem from the relative size differences of the food and digit stimuli. In particular, it could be that the increased size of the food compared to the digit stimulus led to an improved attentional focus on the food stimulus, which improved the reconfiguration of the food task-set, which led to a reduction of the switch costs for the food compared to the digit task.

## Present Study

In this study, we investigated factors contributing to the modulation of cognitive control during task switching with food stimuli. Specifically, the study examined whether the switch-cost asymmetry reported by Maydych et al. (2024) persists when the stimulus size was inverted. In particular, the digit stimuli were presented (always) larger than the food stimuli. This approach allowed us to test whether stimulus size modulates the switch-cost asymmetry with food stimuli. Participants performed two categorization tasks: the classification of food pictures as sweet/savory (food-task) and the classification of digits as odd/even (digit-task). The tasks were performed in a predictable sequence (AABB) using the alternating-runs procedure.

Several possibilities are conceivable for how cognitive control can be modulated in the current investigation. First, if task content drives switch-costs asymmetries, then this should result in a replication of the results reported by Maydych et al. (2024). For that case, we predict that the switch costs, i.e., the RT difference between switch and repetition trials, should be larger for the digit task compared to the food task. This pattern of results would indicate that switch costs increase when participants switch from the food to the digit task compared to the opposite switch direction. This result pattern would suggest that the affective-motivational task properties improve reconfiguration processes for the food compared to the digit task set.

In contrast, if stimulus size determines the modulation of the switch costs, we predict the opposite switch cost pattern compared to the results reported by Maydych et al. (2024). For that case, we predict that the switch costs, i.e., the RT difference between switch and repetition trials, should be larger for the food task compared to the digit task. This pattern of results would indicate that the switch costs increase when participants switch from the digit to the food task, compared to the opposite switch direction. It would further imply that the visual size can facilitate the reconfiguration of task sets, thus reflecting the modulation of cognitive control.

Third, it is conceivable that task content *and* stimulus size jointly modulate switch costs. Accordingly, task content may bias the cognitive system toward a more efficient reconfiguration of one task set over the other, e.g., improved reconfiguration of the food task-set due to motivational relevance compared to the digit task-set. The visual size of the stimuli might further modulate this bias. The variation of the stimulus size could vary the attentional focus on the stimulus, facilitating a reconfiguration process, modulating the switch costs. To investigate this possibility, a direct comparison of the switch cost pattern across the study of Maydych et al. (2024) with the switch cost pattern of the current study is required.

To investigate this possibility, we compared the present data with the control condition reported by Maydych et al. (2024). We therefore first briefly summarize the method, procedure, and key findings of their control condition before turning to a description of the current study.

## Method

### Participants, Material, and Procedure as in Maydych et al. (2024)

Fifty-six participants were assigned to the control condition. Stimuli consisted of digits 1–9 (except 5) and eight food images (four sweet, four savory) chosen from the validated food image database for experimental research on eating and appetite (Blechert et al., 2019). Images used were nos. 85, 145, 387, and 723 (savory), and nos. 6, 78, 139, and 140 (sweet) matched for valence, arousal, complexity, and palatability.

Participants categorized digits (odd/even) and the food pictures (sweet/savory) using “D,” “F,” “J,” and “K” keys on a QWERTZ keyboard. The digit task was mapped to “F” and “J” keys, the food task to “D” and “K,” with task-to-finger mapping counterbalanced across participants.

The stimuli were presented as a compound stimulus, with a smaller digit stimulus presented in gray within white boxes overlaid in the center of the image of the larger food stimulus. On a 24″ monitor and a viewing distance of 80 cm, the white box containing the digit stimulus subtended a visual angle of 1.72° × 1.22°, and the background image containing the food stimulus subtended a visual angle of 12.84° × 10.71°.

Each trial began with a fixation cross displayed for 500 ms, followed by stimulus presentation until response or 3,000 ms. Participants received feedback for 500 ms in the form of “Richtig” or “Falsch” (German for “right” and “wrong”). If no response was made within the allotted time, “Zu langsam” (German for “too slow”) appeared instead. Followed by a 500-ms intertrial interval (ITI). Each food image was paired with every digit, resulting in 64 unique combinations.

Participants completed alternating runs switching, requiring them to switch on every second trial with the predictable task sequence AABB. The experiment included one 64-trial practice block and five 64-trial experimental blocks, with short breaks in between.

### Participants

In the current investigation, a total of 24 (20 female, mean age = 23 years) psychology students from Martin Luther University Halle-Wittenberg, participated. We conducted an a priori power analysis with G*Power 3.1 (Faul et al., 2007) for an interaction between task and trial type, reflecting a modulation of the switch costs on RTs. We assumed an effect size η_G_^2^ = .103 (*f* = .33) for the interaction between task and trial type on RTs (Johnson, 2009; Langsdorf, Kübler et al., 2026; Langsdorf, Schubert et al., 2026).^1^ This resulted in a sample size of 21 participants (α err prob: .05; Power (1 − β err prob): .95). The data collection of *n* = 24 was deemed sufficient to detect the interaction between task and trial type. The experimental protocol conformed to the Declaration of Helsinki, and written informed consent was obtained from each participant before the start of the experiment. All participants were right-handed, German native speakers, and had normal or corrected-to-normal vision. After the experiment, participants were debriefed. The data and scripts are obtainable from https://osf.io/52bxn/.

### Materials

**Figure 1:**
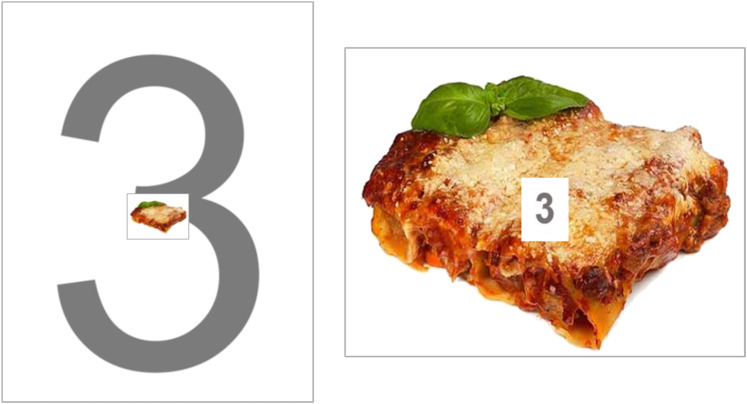
Left: example of a food-digit stimulus as presented in the current experiment. Right: example of a food-digit stimulus as presented in Maydych et al. (2024).

Participants sat in front of a 24″ computer screen with a resolution of 1920 × 1200 pixels, located at eye level with a distance of 80 cm. Stimulus material comprised eight digits from one to nine (except 5) and eight food images (four sweet, four savory). We chose the identical food pictures as Maydych et al. (2024). Participants classified digits (odd/even) and the food stimuli (sweet/savory) with their left middle and index fingers pressing “D” and “F,” and with their right index and middle fingers pressing the keys “J” and “K” on a QWERTZ keyboard. The food task was mapped to “D” and “K” keys, and the digit task to “F” and “J.” The task-finger mapping was counterbalanced across participants. The stimuli were presented as a compound stimulus consistent with the format used by Maydych et al. We maintained a constant area ratio between the white box and the background image. Only exchanging the visual stimulus size of the digit and food stimuli across studies: In the current study, the food stimuli were depicted within the white box subtending a visual angle of 1.72° × 1.22° framed by a thin gray line for figure-ground segregation. Digit stimuli were presented as the background image, subtending a visual angle of 12.84° × 10.71° (Figure 1). All stimuli were presented on a white screen background with a gray frame.

**Figure 2:**
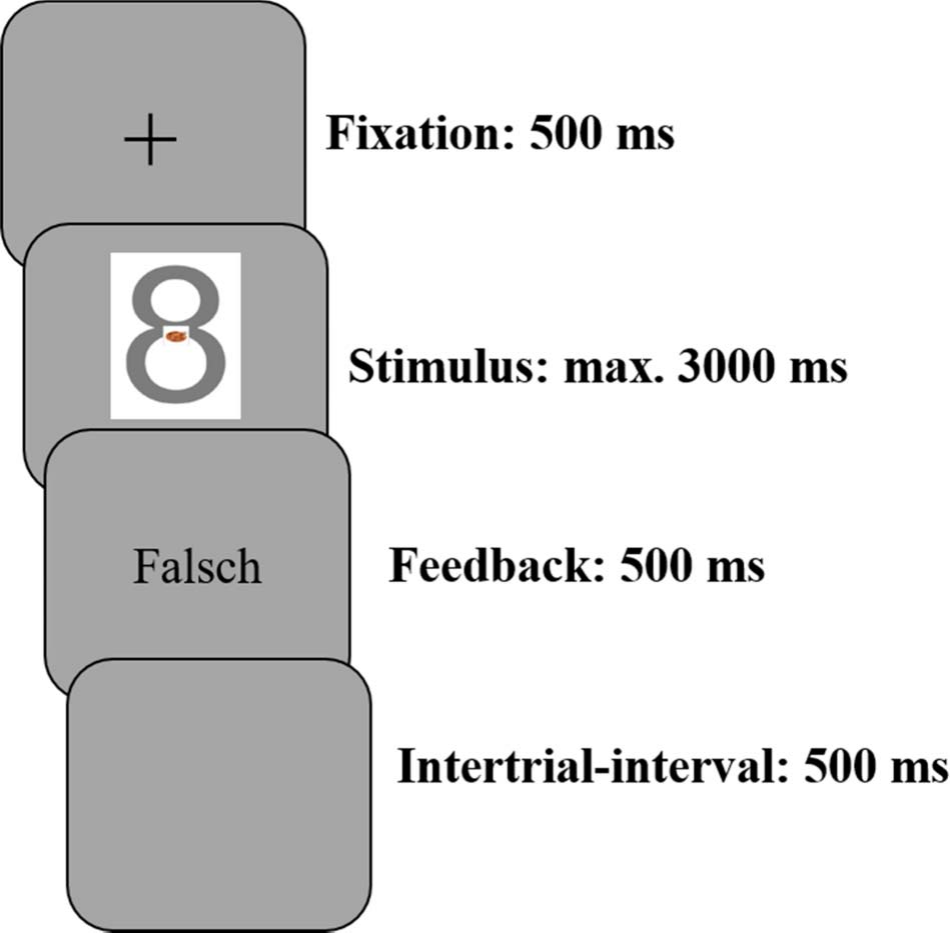
Trial structure. A fixation cross was presented for 500 ms, followed by the food-digit stimulus until a maximum of 3,000 ms elapsed. After feedback for 500 ms, an intertrial interval of 500 ms followed.

Each trial began with a 500-ms fixation cross, followed by the compound stimulus, which remained onscreen until response or a 3,000-ms timeout (Figure 2). Feedback “Richtig,” “Falsch,” or “Zu langsam” (German for “correct,” “wrong,” and “too slow”) was displayed for 500 ms, followed by a 500-ms ITI. Each of the 64 food–digit combinations was presented five times in a random order.

### Procedure

To aid recognition, participants were first provided with printed versions of the food images, which were later presented in a smaller format. Practice blocks with 24 trials were conducted separately for the food and digit tasks. Afterward, participants should repeat and switch between the two tasks in a predetermined order (AABB). Participants completed five experimental blocks, each consisting of 64 trials. Breaks were provided after each block, and participants could resume the experiment at their own pace.

### Data Processing

We subjected the mean RTs and error rates to analyses of variance (ANOVA) with the within-subject factors task (food task, digit task) and trial type (repetition trials, switch trials). A significance threshold of 5% was used. Trials with an erroneous response (*m* = 9.2%) and outliers that deviated more than ± 2.5 *SD*s from mean RTs for each participant and factor combination (*m =* 1.4%) were excluded from the data set. All analyses were conducted in R (R Core Team, 2026).

## Results

### Reaction Times

**Figure 3:**
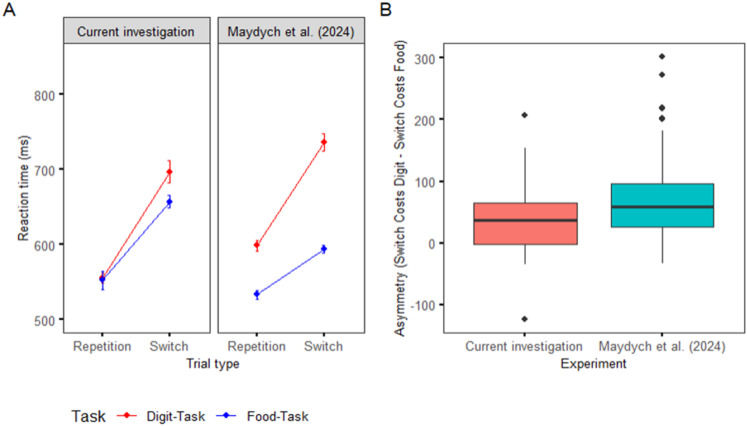
A) Depicts mean reaction times (RTs) as a function of task and trial type for the current study and the study of Maydych et al. (2024). Error bars represent the standard error of the mean. B) Depicts the difference between switch costs (switch–repetition) for each task per experiment. Participants switched faster from the digit toward the food task; stimulus size likely modulated the switch-cost asymmetry across studies.

A detailed analysis of RTs and error rates is reported in Maydych et al. (2024). Briefly, the analysis of the control condition^2^ revealed that task content modulated the switch costs. In particular, RTs were influenced by both the type of task and trial type, as shown by significant main effects of task, *F*(1, 55) = 181.28, *p* < .001, η_p_^2^ = .77, trial type, *F*(1, 55) = 104.59, *p* < .001, η_p_^2^ = .66, and a significant interaction between the two factors, *F*(1, 55) = 52.25, *p* < .001, η_p_^2^ = .49 (see Figure 3A). The switch costs were decreased for the food task (*m* = 60 ms) compared to the digit task (*m* = 137 ms), *t*(55) = −7.23, *p* < .001, *d* = −0.97. Participants switched faster from the digit to the food task compared to the opposite switch direction. These findings are consistent with the assumption that reconfiguration of the food compared to the digit task was processed more efficiently, potentially due to its motivational saliency.

Concerning the current results, we were interested in whether a similar switch cost pattern emerges when visual salience is inverted in the food and digit tasks across studies. We obtained a significant main effect of the factor trial type on RTs, *F*(1, 23) = 105.17, *p* < .001, η_p_^2^ = 0.82. Participants responded faster in repetition trials (*m* = 536 ms) than in switch trials (*m* = 658 ms), reflecting switch costs (Rogers & Monsell, 1995). Most importantly, the interaction between the factors trial type and task reached significance, *F*(1, 23) = 7.01, *p* < .014, η_p_^2^ = 0.23. Switch costs were decreased for the food task (*m* = 105 ms) compared to the digit task (*m* = 141 ms; Figure 3A), in line with the results by Maydych et al. (2024). The factor task did not reach significance, *F*(1, 23) = 2.58, *p* = .122, η_p_^2^ = 0.100. Results across both investigations suggest a similar pattern of switch costs, with reduced switch costs for the food compared to the digit task, indicating an effect of task content on cognitive control processes.

### Error Rates

An analysis of the error rates in the control condition of Maydych et al. (2024) revealed an effect of the factor task, *F*(1, 55) = 65.42, *p* < .001, η_p_^2^ = .54, trial type, *F*(1, 55) = 32.11, *p* < .001, η_p_^2^ = .37, and a significant interaction between both factors, *F*(1, 55) = 13.66, *p* < .001, η_p_^2^ = .20. Further testing revealed that the switch costs were decreased for the food task (*m* = 1%) compared to the digit task (*m* = 5%*)*, *t*(55) = −3.69, *p* < .001, *d* = −0.49. The results on the error rates indicate that participants switched more accurately from the digit to the food task than in the opposite direction.

For the current results, an analysis of error rates revealed a significant main effect of the factor trial type, *F*(1, 23) = 4.59, *p* < .043, η_p_^2^ = 0.16. Participants made more errors in the switch (*m =* 10%) compared to the repetition condition (*m* = 8%), reflecting switch costs. Furthermore, the factor task reached significance, *F*(1, 23) = 12.49, *p* < .002 η_p_^2^ = 0.35. Participants made more errors executing the digit (*m* = 11%) compared to the food task (*m* = 7%). The interaction between the factors trial type and task also reached significance, *F*(1, 23) = 17.09, *p* < .002 η_p_^2^ = 0.43. The switch costs in error rates were greater for the digit task (*m* = 5%) compared to the food task (*m* = −2%), indicating a similar switch costs pattern in the error rates across studiesTable 1). 

**Table 1:** Mean rates of errors in % (and standard error of the mean) from the current experiment and Maydych et al. (2024) as a function of trial type and task

Task	Current experiment		Maydych et al. (2024)	
	Trial type		Trial type	
	Repetition trials	Switch trials	Repetition trials	Switch trials
Digit task	8.7 (0.8)	13.9 (1.1)	6.5 (0.5)	4.0 (0.4)
Food task	8.1 (0.8)	6.4 (1.0)	11.3 (0.6)	5.0 (0.5)

#### Cross-Study Comparison

We directly compared the switch cost pattern between Maydych et al. (2024) and the current study, testing whether a similar switch cost pattern emerged when the digit stimulus was visually salient, relative to the food stimulus. We applied a mixed-design ANOVA with task (food task, digit task) and trial type (repetition trials, switch trials) as within-subject factors, and experiment (Maydych et al., current investigation) as a between-subject factor. We first report the results on RTs, followed by the error rates. To avoid redundancy, we only report statistical results that involve the factor experiment. For follow-up comparisons, we conducted Welch´s *t*-tests to account for unequal sample sizes and possible variance differences between samples (West, 2021).

The analysis yielded a significant interaction between the factors experiment and task on RTs, *F*(1, 78) = 31.94, *p* < .001, η_p_^2^ = 0.29. While neither the main effect of the factor experiment, *F*(1, 78) = 1.72, *p* = .194, η_p_^2^ = 0.02, nor the interaction between the factors experiment and trial type reached significance, *F*(1, 78) = 2.11, *p* = .150, η_p_^2^ = 0.03.

Most relevant, we obtained a significant interaction between the factors experiment, task, and trial type on RTs, *F*(1, 78) = 4.61, *p* < .035 η_p_^2^ = 0.06, reflecting a modulation of the asymmetric switch costs across experiments. Across both studies, the switch costs were consistently greater for the digit task compared to the food task. In the study of Maydych et al. (2024), compared to the current investigation, the switch costs for the digit task were of a similar degree (137 ms vs. 141 ms), *t*(63.22) = 0.21, *p* = .832, *d* = 0.04. While the switch costs for the food task were decreased in the study of Maydych et al. (2024) compared to the current investigation (60 ms vs. 105 ms), *t*(34.41) = 3.06, *p* < .004, *d* = 0.84. Despite quantitative differences in the degree of asymmetry in switch costs, both studies consistently show greater switch costs for the digit task relative to the food task.

This is illustrated in Figure 3B, in which the degree of asymmetry in the switch costs is illustrated for both studies. To quantify the degree of asymmetry, we computed the switch cost difference (digit task switch costs minus food task switch costs) for both studies: 77 ms in the study of Maydych et al. (2024) compared to 37 ms in the current investigation, *t*(50.51) = −2.29, *p* < .03, *d* = −0.52. Overall, the direction of the asymmetry was consistent, while its magnitude was modulated by the visual size of stimuli. These results provide initial evidence for the assumption that food stimuli improve cognitive control, while visual stimulus size further seems to modulate this effect.

The analysis of error rates across studies revealed only a significant main effect of the factor experiment, *F*(1, 78) = 6.12, *p* < .016, η_p_^2^ = 0.07. The interactions between experiment and task, experiment and trial type, and the three-way interaction with experiment, task, and trial type were all nonsignificant (all *ps* > .11). These results indicate that visual stimulus size was not modulating the accuracy of cognitive control.

## Discussion

In this study, we investigated whether food stimuli, the size of stimuli, or both factors modulate cognitive control, as reflected by switch-cost asymmetries. To test this, participants repeated and switched between a food and a digit classification task. We obtained typical switch costs, with slower responses on switch compared to repetition trials. Most importantly, and consistent with Maydych et al. (2024), we obtained a switch-cost asymmetry across studies, with greater switch costs for the digit than for the food task. However, this asymmetry was attenuated in the current study compared to Maydych et al., suggesting that while task content robustly modulates cognitive control, stimulus size can modulate the magnitude of this effect. The results support the assumption that food stimuli bias cognitive control in task switching and that visual size potentially further tunes this effect.

How can we interpret the modulation of cognitive flexibility by food stimuli? It is conceivable that the motivational saliency of the food stimuli leads to a rapid activation of the food task-set compared to the digit task-set in working memory. This rapid activation should result in reduced switch costs for the food compared to the digit task and can reflect a more efficient reconfiguration of the food compared to the digit task-set (Rutters et al., 2015). This activation process might be further improved by increased stimulus size, leading to improved attentional focus on the stimulus and resulting in the modulation of the switch-cost asymmetry across studies.

Next, we consider the differences between the present findings and those reported by Maydych et al. (2024). In that study, responses in the food task were faster than in the digit task, whereas this advantage was absent in the current investigation. Moreover, switch costs for the food task were smaller in Maydych et al. than those observed here.

Mutually nonexclusive explanations may account for these differences. First, the reduction in stimulus size may have directly reduced perceptual processing efficiency. Smaller compared to larger food stimuli may require prolonged processing; this size reduction could, in turn, have attenuated the relative processing advantage of the food compared to the digit task. This relatively prolonged processing can have led to the increased switch costs in the current study compared to Maydych et al. (2024).

Second, the size reduction across studies may have reduced the motivational saliency of the food stimuli. Prior work suggests that stimulus size can modulate motivational saliency (Codispoti & De Cesarei, 2007; De Cesarei & Codispoti, 2010). If reducing stimulus size diminished the motivational saliency of the food stimulus, then, as a side effect, this could have attenuated the influence of the food information on further processing and, consequently, on the cognitive control.

Overall, future research could aim to disentangle whether the observed differences are related to changes in perceptual processing efficiency and/or in motivational salience. Importantly, the direction of the switch-cost asymmetry was consistent across studies, which indicates that stimulus size alone is unlikely to be the sole determinant of the observed switch-cost asymmetry.

Let us turn to the findings of Maydych et al. (2024) to further contextualize the present results. The authors reported that the switch-cost asymmetry between tasks was boosted under a fasting compared to a control condition, which indicates that the motivational state of participants and the properties of the food stimulus are jointly modulating the control processes. This boosted switch-cost asymmetry is explainable with the assumption that this effect may, at least in part, be caused by the motivational saliency of the food stimulus. Accordingly, the food stimuli may bias processing so that participants are drawn toward them. This could have caused the reduced switch costs for the food task, in the case when participants switch toward the food stimuli. Future research may focus on elucidating how this modulation is achieved, e.g., by reducing between-task interference.

Additionally, further characteristics of the present task material may have theoretically contributed to the obtained effects. For example, differences in the coloration between the food and digit stimuli may have contributed to the observed results. Classic research on attentional control indicates that colored stimuli can enhance attentional capture. However, more recent evidence suggests that the influence of features such as color is not mandatory, as salient perceptual features can be actively suppressed when they are task-irrelevant (Huang & Li, 2023). Moreover, if color processing had substantially contributed to the results, one should have expected a strong processing advantage for the food compared to the digit task, given that the stimuli were presented foveally, where cone density, and thus sensitivity to chromatic information, is higher than for the gray-colored digit stimuli. Since the classification of food stimuli was not faster than that of digit stimuli, we conclude that although differences in coloration cannot be excluded as a factor potentially influencing the processing of food and digit stimuli, it is not likely that this can explain the findings of asymmetric switch cost as a sole account. This is further underscored by the finding of the fasting-related boosting of the switch-cost asymmetry by Maydych et al. (2024).

Importantly, we kept the paradigm closely comparable to that of Maydych et al. (2024) to directly test whether the previously reported switch-cost asymmetry persists when the size confound in that study is controlled. Thus, the present study primarily addressed this methodological limitation while preserving the core structure of the original paradigm. Importantly, the direction of the switch-cost asymmetry remained stable across studies, indicating a robust effect of task content on control processes. These findings suggest that stimulus size did not reverse the direction of the switch-cost asymmetry, nor did other potential confounds. Future studies should examine additional relevant factors (e.g., stimulus types) that may contribute to this effect.

### Conclusion

We provided initial evidence for the assumption that task content modulates cognitive control as reflected in switch costs, while visual stimulus size possibly further modulates it. Converging evidence favors the assumption that food stimuli can facilitate cognitive control, while indicating the importance of considering the stimulus size when using complex stimuli.

## Footnotes

^1^
These studies used face stimuli to investigate switch-cost asymmetries, which may have produced stronger modulation of control processes.

^2^
Here, we report only the results from the control group (*n* = 56), which have not previously been reported separately, and used this group for a cross-study comparison with the present findings.

## Notes

**Publication Ethics:** The experimental protocol conformed to the Declaration of Helsinki, and written informed consent was obtained from each participant before the start of the experiment.

**Open Science:** To the best of my ability and knowledge, I have provided all original materials and clear references to all other materials via a stable online repository.

Open Data: The data and scripts are obtainable from https://osf.io/52bxn/ (Langsdorf, Schubert, et al., 2026).

Open Materials: The materials are obtainable from https://osf.io/52bxn/ (Langsdorf, Schubert, et al., 2026).

Preregistration: Our manuscript contains no experiment with a completely executed preregistration.
